# Ion mobility mass spectrometry enhances low-abundance species detection in untargeted lipidomics

**DOI:** 10.1007/s11306-016-0971-3

**Published:** 2016-02-08

**Authors:** Abdul Basit, Silvia Pontis, Daniele Piomelli, Andrea Armirotti

**Affiliations:** Department of Drug Discovery and Development, Istituto Italiano di Tecnologia, via Morego 30, 16163 Genoa, Italy; Departments of Anatomy and Neurobiology, Pharmacology and Biological Chemistry, University of California, Irvine, CA 92697 USA

**Keywords:** Untargeted lipidomics, Ion-mobility mass spectrometry, Neuroinflammation, *N*-acyl phosphatidylethanolamines

## Abstract

We describe a simple method for the detection of low intensity lipid signals in complex tissue samples, based on a combination of liquid chromatography/mass spectrometry and ion mobility mass spectrometry. The method relies on visual and software-assisted analysis of overlapped mobilograms (diagrams of mass-to-charge ratio, *m/z*, *vs* drift time, DT) and was successfully applied in untargeted lipidomics analyses of mouse brain tissue to detect relatively small variations in a scarce class of phospholipids (*N*-acyl phosphatidylethanolamines) generated during neural tissue damage, against a background of hundreds of lipid species. Standard analytical tools, including Principal Component Analysis, failed to detect such changes.

**Electronic supplementary material:**

The online version of this article (doi:10.1007/s11306-016-0971-3) contains supplementary material, which is available to authorized users.

## Introduction

Untargeted liquid chromatography/mass spectrometry (LC/MS)-based analyses are widely used to profile the lipidome of complex biological samples and to generate testable hypotheses about the functions served by specific lipid pathways in health and disease (Han et al. 2011; Astarita and Piomelli 2011; Armirotti et al. 2014). By its very nature, this type of analyses requires the acquisition of large amounts of high-resolution MS data, typically collected over a period of tens of minutes, and produces thousands of individual data points that must be extracted from total ion currents and compared across multiple sample replicates. From such substantial data sets, biologically relevant information may be best obtained using unsupervised data analysis procedures such as Principal Component Analysis (PCA) (van den Berg et al. 2006; Ramadan et al. 2006), which has become a standard post-acquisition tool in untargeted metabolomics (Blasco et al. 2013). Despite their proven usefulness, PCA-based untargeted lipidomics analyses are known to overlook rare lipid species, which very often include molecules of considerable biological importance (e.g., lipids involved in cellular signaling). As a rule, therefore, such low-abundance lipids are investigated using targeted LC-MS/MS protocols. In the present study, we asked whether IMS—a technique initially developed for the detection of trace quantities of gaseous organic compounds but recently applied with success to metabolomics (Baker et al. 2014)—might help to heighten the sensitivity of detection for scarce lipid species in complex biological matrices. IMS adds an additional gas-phase separation to LC/MS analysis (May et al. 2015). This separation depends on the collisional cross sections of metabolites (CCS) that closely correlate with their chemical structure. CCS has been recently proposed as a tool to support metabolite identification (Paglia et al. 2015). IMS also enables the exploration of composite data sets using mobility-filtered ion maps—that is, plots of *m/zversus* DT values, also known as mobilograms—that can be subsequently interrogated using dedicated search tools. Such tools allow investigators to overlap mobilograms from different samples, selectively highlighting with pseudocolors of variable intensity those ions that are differentially represented in the samples (comparative mobilogram analysis, CMA). To assess the usefulness of CMA in untargeted LC/MS-based lipidomics, we explored the very early stages of a neuroinflammatory process by injecting 6-hydroxy-dopamine (6-hydroxy-DA) in the dorsal striatum of mouse brain and we performed untargeted lipidomics analyses 48 h later, when no overt functional alterations were yet observed. Our objective was to probe whether CMA may help to detect small changes in low abundance lipids that might be biologically related to the developing pathology.

## Materials and methods

### Reagents, standards, instruments and software

Solvents and chemicals were purchased from Sigma Aldrich (Saint Louis, MO, USA). Unless otherwise indicated, all LC-MS instruments, columns and software were from Waters Inc. (Milford, MA, USA).

### Animal handling and 6-hydroxy-DA administration

Male 8–10 weeks old mice were anesthetized with a mixture of ketamine/xylazine (100 and 10 mg/kg body weight, respectively) and placed in a stereotaxic frame with a mouse-adaptor (Stoelting, Wood Dale, USA). 6-hydroxy-DA was dissolved at a concentration of 3.2 μg/μL of ice-cold 0.9 % saline solution containing 0.02 % ascorbate. Two injections of 1 μL each were made at the following brain atlas coordinates (in mm relative to bregma and dural surface, Paxinos and Franklin 2001): (i) AP = +1.0, L = −2.1, DV = −2.9; and (ii) AP = +0.3, L = −2.3, DV = −2.9. Sham lesions were carried out by 1 μL injection of 0.02 % ascorbic acid-saline at the same coordinates. All procedures were performed in compliance with Italian regulations on protection of animals used for experimental and other scientific purposes (D.M. 116192) as well as with European Economic Community regulations (O.J. of E.C. L 358/1 12/18/1986). Forty-eight hours after 6-hydroxy-DA injection, mice were anesthetized with chloral hydrate (450 mg/kg) and killed by decapitation; the brain were rapidly removed and dorsal striatum and substantia nigra were dissected, flash frozen and stored at −80 °C.

### Immunofluorescence

Mice were anesthetized with chloral hydrate (400 mg/kg), and transcardially perfused with 20 mL of 0.9 % saline solution followed by 60 mL of 4 % paraformaldehyde in saline. Tissue was post fixed in paraformaldehyde 4 % for 1 h and then stored in 30 % sucrose for 3 days. Forty micrometer sections, one every fifth, were collected and processed for immunohistochemistry. Sections were incubated with anti Iba1 (Wako, Osaka) primary antibody followed by the secondary antibody Alexa fluor 488 (Life science, USA). Images were collected with a Nikon A1 confocal microscopy with a 60X 1.4 numerical aperture objective lens.

### Sample preparation

Brain tissue, collected from 10 mouse brains, was transferred to pre-weighted 7 mL glass vials. Wet tissues were then weighed and homogenized in chloroform:methanol (1:2; vol/vol), added to each vial using a 1 mL/5 mg wet tissue ratio. After mixing for 30 s with a Vortex^®^, chloroform (0.3 mL/5 mg tissue) and water (0.3 mL/5 mg tissue) were sequentially added and mixed after each addition. The samples were then centrifuged for 15 min at 3500×*g* at 4 °C. The organic phases (lower fractions) were transferred to glass vials. To increase the overall recovery, the aqueous phase (upper fraction) was re-extracted with chloroform (0.5 mL/5 mg tissue). The two resulting organic phases were pooled, evaporated under N_2_ and the residue was dissolved in methanol/chloroform (9:1, vol/vol; 0.1 mL/10 mg tissue). After mixing for 30 s and centrifugation for 10 min at 5000×*g*, 4 °C), the samples were transferred to glass vials for analyses.

### Untargeted LC-IMS

Samples were loaded onto an Acquity UPLC system coupled to a SynaptG2 QToF mass spectrometer. Lipids were separated on a reversed-phase CSH column (2.1 × 100 mm, 1.8 µm) and eluted at 0.4 mL/min using the following gradient conditions: A = 10 mM ammonium formate in 60:40 acetonitrile/water, B = 10 mM ammonium formate in 90:10 isopropyl alcohol/acetonitrile; after 1 min at 30 %, solvent B was brought to 45 % in 1 min and maintained for 1 min, then to 50 % in 6 min, and then to 100 % in 9 min, followed by a 1 min 100 % B isocratic step and reconditioning to 30 % B. Total run time was 21 min. Injection volume was set to 2 μL. Each sample was acquired in triplicate. The MS instrument was operated in both positive and negative ion modes, setting the capillary and the cone voltages at 3 kV and 35 V for ESI^+^ and 2.2 kV and 40 V for ESI^−^ respectively. The source temperature was 90 °C. Desolvation and cone gas flows (N_2_) were set to 800 and 20 L/h respectively. Desolvation temperature was set to 400 °C. Ion mobility parameters were set as follows: wave velocity and wave height were set to 600 m/s and 40 V respectively, He cell and IMS cell gas flows were set to 180 and 90 mL/min. respectively. Data were acquired in both MS^e^ and MS/MS mode (Pedersen et al. 2013; Yilmaz et al. 2013) with fragmentation performed in both the trap and the transfer regions, with the latter method allowing parent-to-fragments realignment by IMS (fragments are generated after the ion mobility separation and thus they share the same drift time as their parents). Low energy scans were acquired at fixed 4 eV potential and high energy scans were acquired with an energy ramp from 25 to 45 eV. Scan rate was set to 0.3 s per spectrum. Scan range was set to 50 to 1200 *m/z*. Leucine enkephalin (2 ng/mL) was infused as lock mass for spectra recalibration.

### NAPE synthesis

NAPEs were synthesized following the protocol described by Guo and colleagues (Guo et al. 2010). Briefly, 0.025 g (0.03 mmol) of 1-stearoyl-2-docosahexanoyl-*sn*-glycero-3-phosphoethanolamine was dissolved in 2.5 mL chloroform containing 0.0125 g triethylamine in a precooled 7 mL glass vial. An appropriate acyl chloride (16:0, 17:0 or 18:0, 0.12 mmol each) in 0.9 mL chloroform was added dropwise. After the addition was complete, the reaction mixtures were heated to 40 °C for 2 h and stirred overnight at room temperature. The reactions were quenched by adding a saturated sodium bicarbonate solution (2.5 mL), and the organic layers were collected and washed with 3.0 mL of 0.01 M hydrochloric acid and 3.0 mL of brine. The reaction mixtures were dehydrated with sodium sulfate and the product was dried in a N_2_ evaporator and purified using silica gel chromatography. The purified NAPEs were weighed and the final amount of NAPE obtained (in mg) was compared with the theoretical one. The reaction yield ranged from 75 to 80 %.

### Targeted NAPE analysis

The brain samples were re-extracted using the above synthetic exogenous 18:0-22:6-N17:0 NAPE to a final 0.5 μM concentration to the methanol/chloroform extraction mixture. Targeted analysis of the samples was then carried out on an Acquity UPLC system coupled with a Xevo TQ-MS triple quadrupole mass spectrometer. NAPEs were separated on a HSS T3 C18 column (2.1 × 50 mm, 1.7 µ) at a flow rate of 0.4 mL/min. The mobile phase consisted of 10 mM ammonium formate (pH 5) in acetonitrile/water (60:40 v/v) as solvent A and 10 mM ammonium formate (pH 5) in acetonitrile/ isopropyl alcohol (10:90 v/v) as solvent B. A linear gradient program was developed for the separation of all metabolites: 0.0–0.5 min 50 % B, 0.5–3.5 min 50 to 100 % B and 3.5–4.5 min maintained at 100 % B. The column was then reconditioned to 50 % B for 1.0 min. The total run time for analysis was 6 min, and the injection volume was set to 5 μL. The mass spectrometer was operated in the positive ESI mode and analytes were quantified using the multiple reaction monitoring parameters indicated in Supporting Table 3. The capillary voltage was set at 3 kV. The cone voltage and collision energy values were set to 25 V and 20 eV respectively for all transitions. The source temperature was set to 120 °C. Desolvation gas and cone gas (N_2_) flows were set to 800 and 20 L/h, respectively. Desolvation temperature was set to 450 °C. This method was validated as already described (Basit et al. 2015): exogenous 18:0/22:6/N17:0 NAPE was spiked in naive brain homogenate and extracted (N = 3). Limit of quantification, recovery, matrix effect, extraction efficiency were evaluated using a calibration curve prepared with the same standard in the 0.05–50 nM concentration range.

### Data analysis

Data were analyzed the using MarkerLynx software: raw data from high-resolution LC-MS/MS runs for either polarity (ESI^+^ or ESI^−^) were subjected to a Pareto-scaled PCA. Accurate masses (*m/z*) and retention time values (RT) were included in the multivariate analysis and assigned as X-variables (markers). Only markers observed in at least 75 % of the experimental replicates were retained. HDMS Compare^®^ software was used to overlap ion mobility/drift time maps from different runs. Three replicates per samples were analyzed by the software. A visual inspection of the regions showing differentially expressed metabolites was then performed. A region of interest, showing clear alterations in the fusion map and spanning from 900 to 1100 *m/z* and 10 to 60 ms drift time, was further investigated by the software and differential chromatograms and mass spectra were calculated and reported. The corresponding *m/z* values were then manually extracted from the original LC-MS chromatograms for confirmation and further inspection. Tentative but unsuccessful lipid ID was carried out by interrogating the METLIN (Smith et al. 2005; Tautenhahn et al. 2012), HMDB (Wishart et al. 2009, 2013) and LipidMaps (Fahy et al. 2009; Schmelzer et al. 2007) databases. Tolerance on *m/z* values was set to 5 ppm. Identification was then based on tandem mass analysis, by means of manual interpretation of the fragmentation pathways, further confirmed by fragment ions accurate mass calculation and comparison with reported literature on NAPEs (Astarita et al. 2008). Further MS and MS/MS data processing and targeted quantification of NAPEs were carried out using MassLynx and TargetLynx softwares. Statistical analysis of NAPE upregulation was performed using GraphPad Prism 5 (GraphPad Software, La Jolla, CA, USA). Data were analyzed using the Student’s *t* test, comparing control and lesioned groups. A *P* value <0.05 was considered significant.

## Results and discussion

**Fig. 1:**
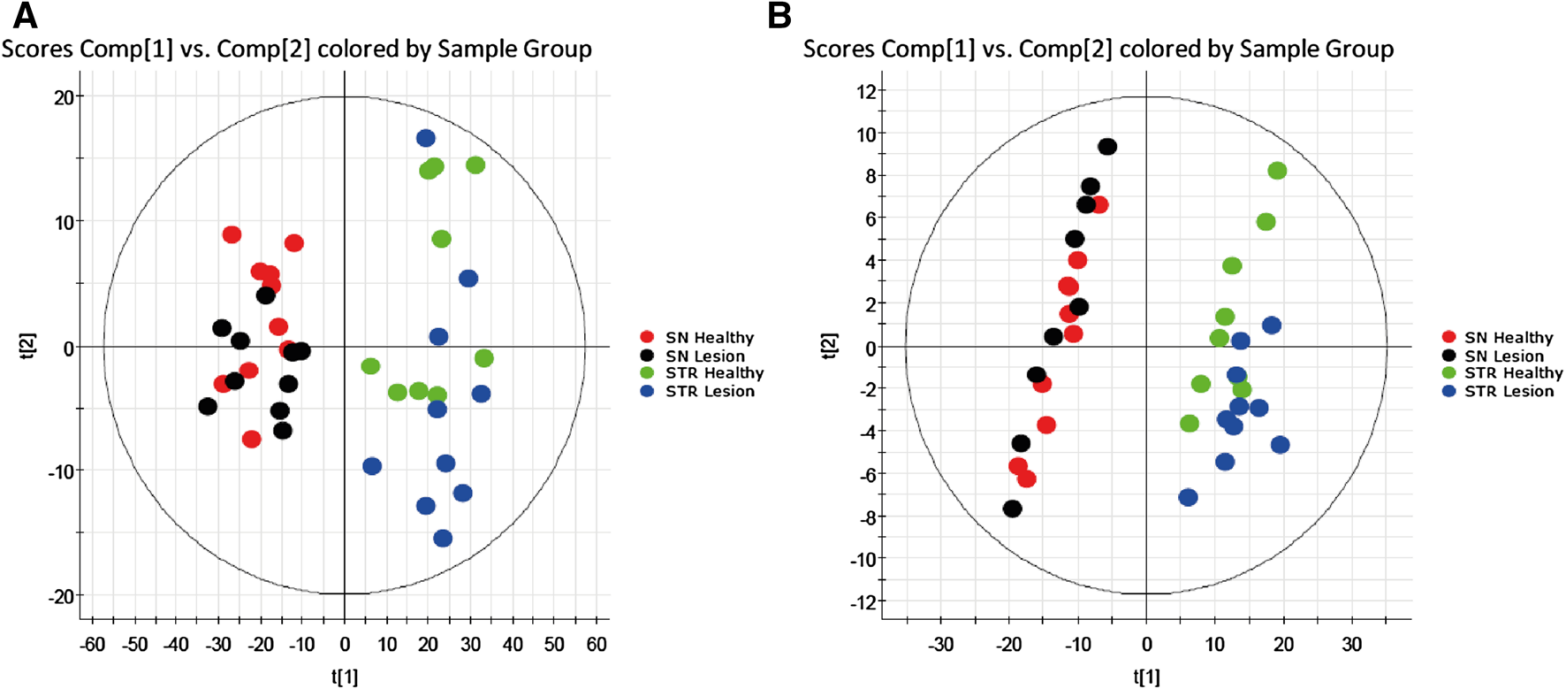
Principal Component Analysis score plots of untargeted lipidomics dataset for ESI^+^ and ESI^−^ modes (*Panels A and B* respectively). The data reveal tissue (substantia nigra vs striatum) but no pathology-related clustering

**Fig. 2:**
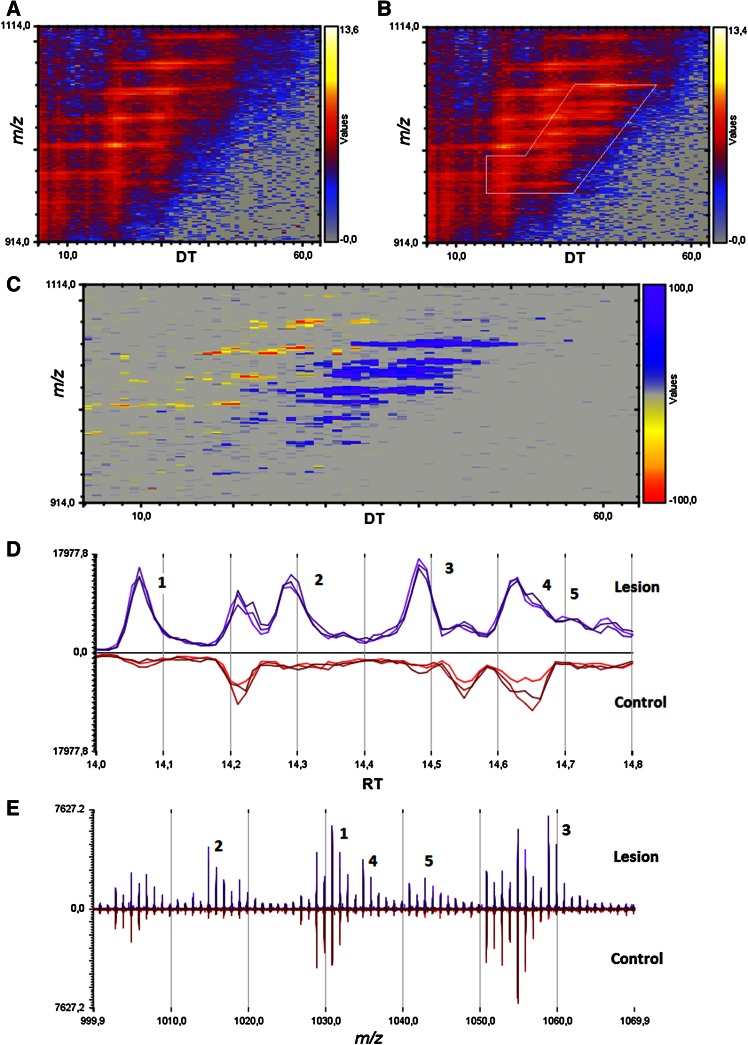
Mobilogram analysis of the same dataset reported in Fig. 1. *Panels A and B*: DT versus *m/z* mobilograms of lesioned (*A*) and control (*B*) striatal tissue. *Panel C* fusion map obtained by overlapping control (*yellow to red*) and lesion (*blue to violet*) tissue mobilograms. A well-defined family of signals (*m/z* = 1000–1100) is highlighted in violet (elevated in lesioned tissue). In *Panel* *B*, the corresponding signals are delimited by a white polygon. *Panel D* differential LC-MS chromatogram showing 5 peaks unequivocally elevated in lesioned striatum. *Panel E* differential mass spectrum showing the corresponding upregulated *m/z* values

**Fig. 3:**
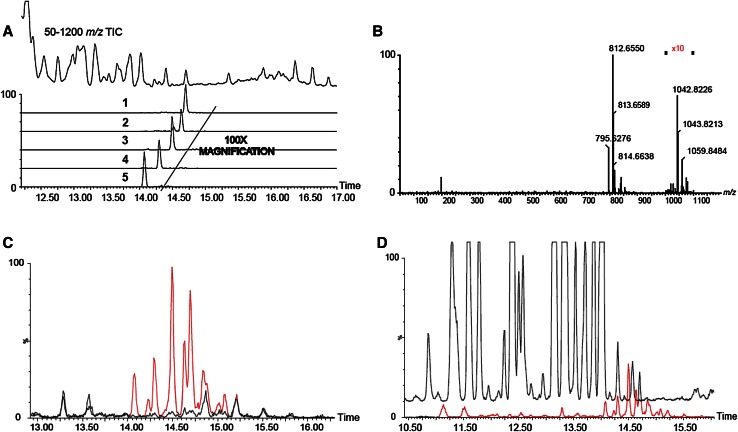
*Panel A* The extracted ion currents of the 5 signals described in Fig. 2 magnified 100 folds to become visible when compared to the corresponding 50–1200 *m/z* total ion current (*upper trace*). *Panel B* representative mass spectrum of molecule 5 (1042.82 m/z, ESI^+^). The corresponding peak is magnified tenfolds. *Panel C* overlapped extracted ion traces of fragment ion *m/z* = 310.31 from control (*black trace*) and lesioned hemisphere (*red trace*). *Panel D* overlapped extracted ion traces of fragments *m/z* = 310.31 (*red*) and a fragment ion of sphingoid base (*m/z* = 246.26 d18:1; *black trace*)

**Fig. 4:**
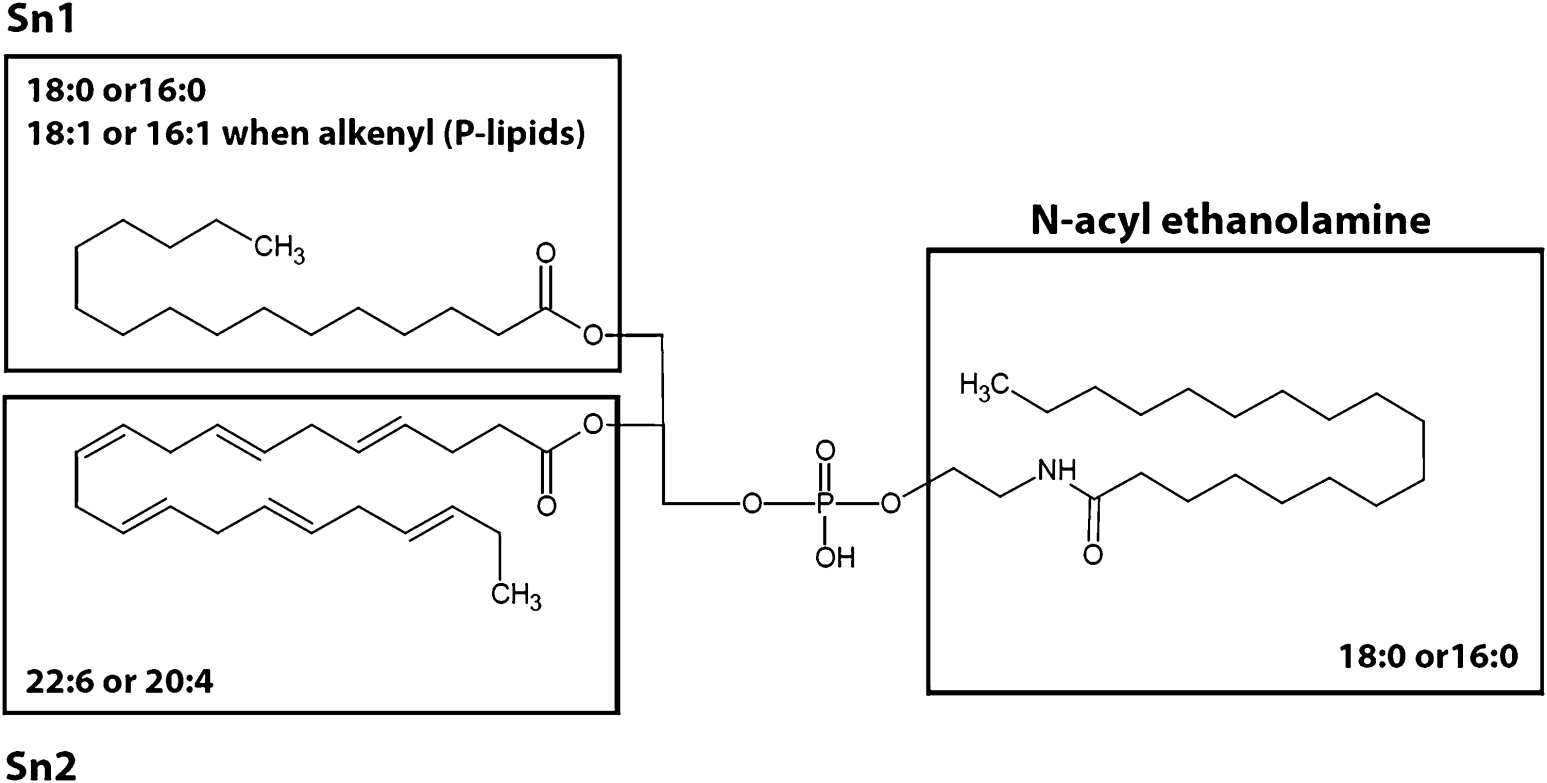
Fatty acyl chain composition of the observed NAPEs

**Fig. 5:**
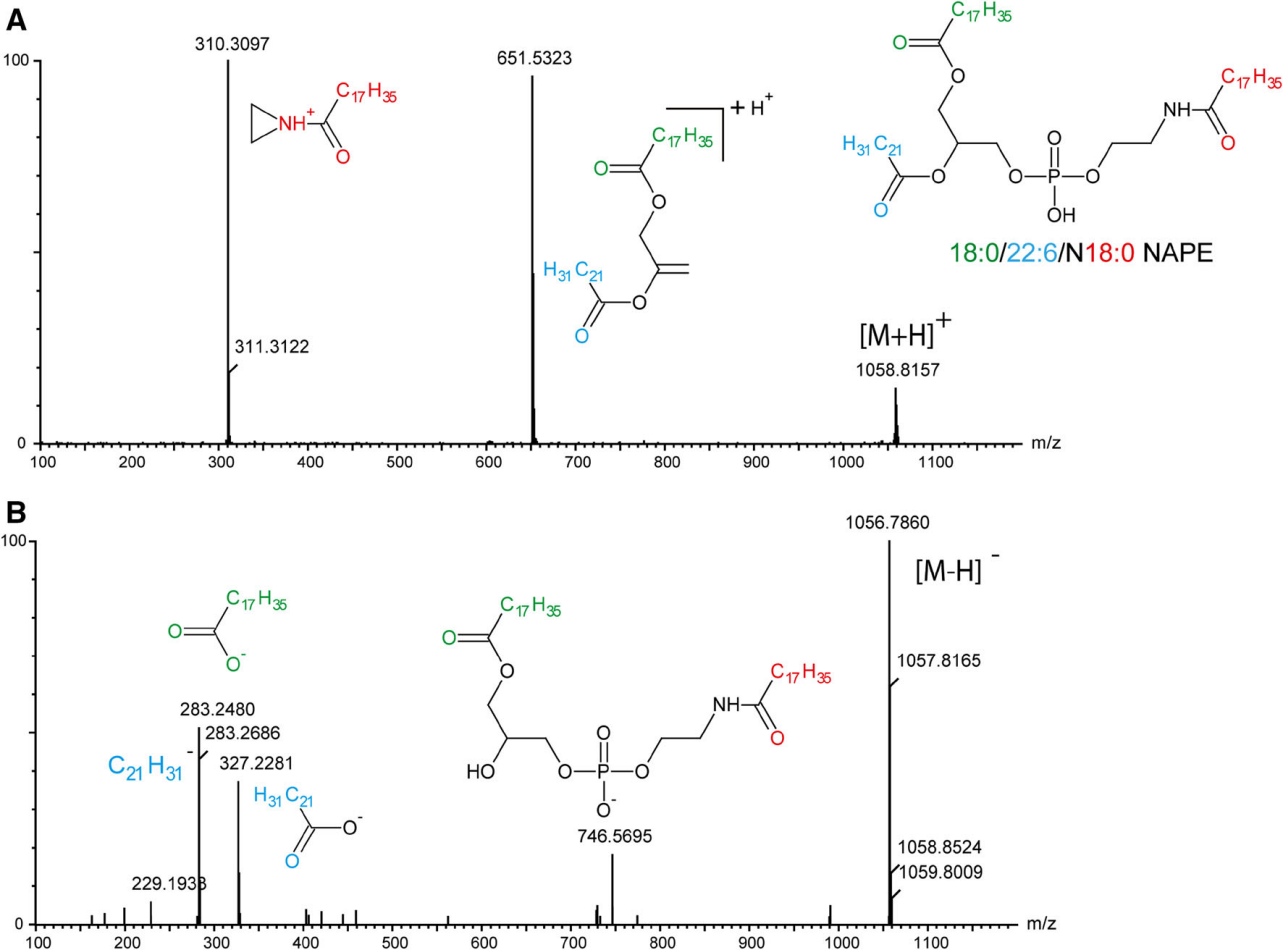
Tandem mass spectra analyses of brain-derived NAPE (18:0/22:6/N18:0) under ESI^+^ (*Panel A*) and ESI^−^ (*Panel B*) conditions

**Fig. 6:**
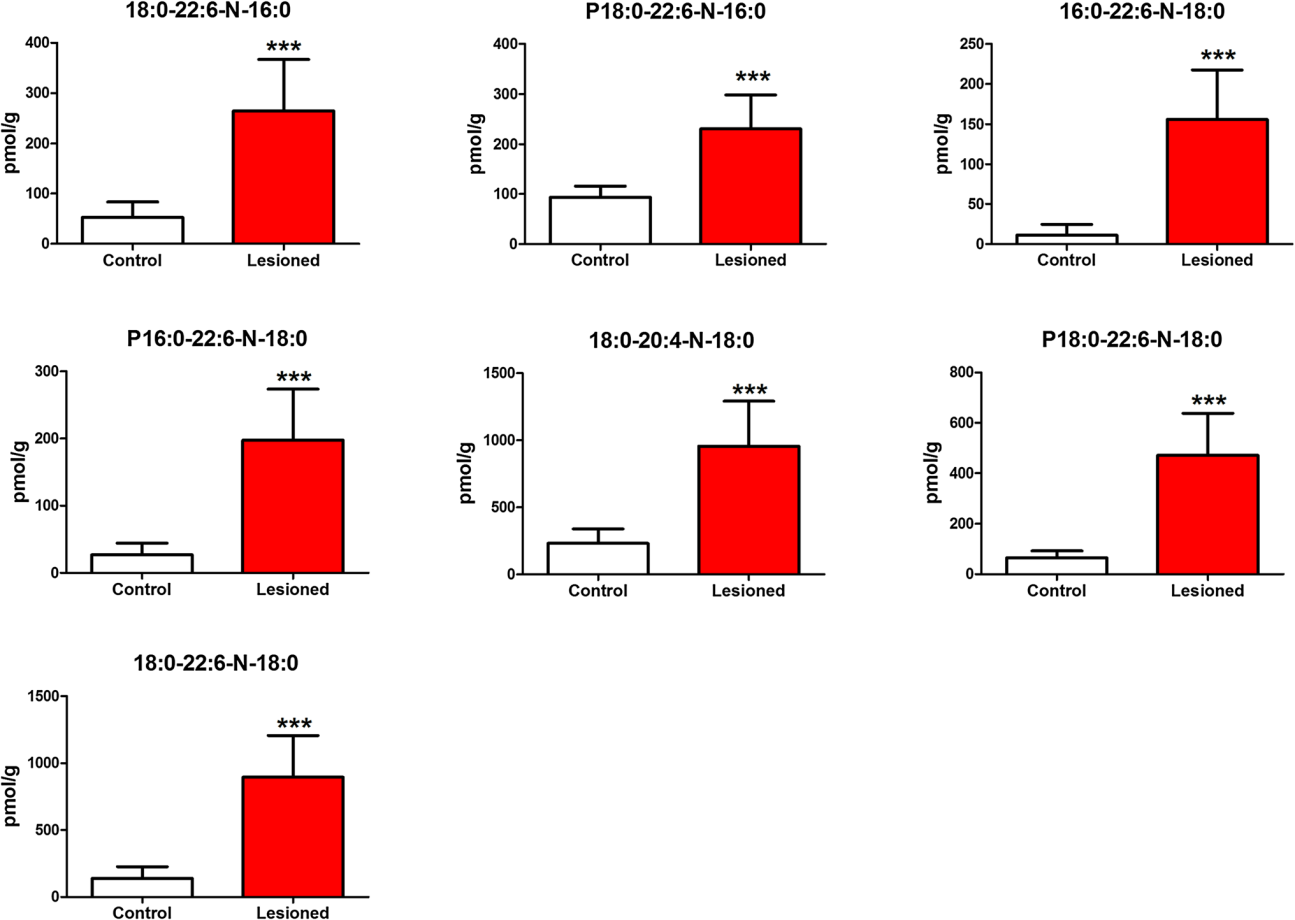
NAPE levels in control (*empty bar*) and lesioned (*red bar*) mouse striatum. ***P < 0.01, 2 tailed *t* test, N = 10 per group

We injected 6-hydroxy-DA, a toxic compound that selectively destroys dopaminergic axon terminals (Morales et al. 2015, Le et al. 2014), into the striatum of mice and, 48 h later, sacrificed the animals, snap-froze their brains, and collected two target regions—striatum and substantia nigra—for lipid analysis. The substantia nigra was included because it contains the cell bodies of the striatal dopaminergic terminals damaged by 6-hydroxy-DOPA (Blesa and Przedborski 2014). Samples of striatum and SN taken from the left (control) and right (lesioned) hemispheres from 10 mouse brains were collected separately, producing a total of 4 experimental groups. Following untargeted high-resolution LC-MS/MS, the data were analyzed using PCA. The score plot reported in Fig. 1 suggests the existence of regional differences in lipid composition between striatum and SN, but does not reveal any statistically detectable change related to 6-hydroxy-DOPA treatment. Nevertheless, immunohistochemical experiments revealed the presence of detectable neural damage in the striatum (as assessed by measuring microglia cell activation, see Supplementary Fig. 1). Because injury and inflammation have been associated with alterations in multiple low-abundance lipid species in the brain (Esposito et al. 2014; Hansen et al. 2000), this negative finding highlights the difficulty in detecting such species using standard PCA-based lipidomics protocols. The same set of samples was then re-acquired in the IMS mode and dedicated software (HDMS Compare^®^) was used to compare the profiles of lesioned and unlesioned striatal tissue. The HDMS Compare^®^ software was originally designed to support the characterization of complex organic materials—including mineral oils, fuels, polymers and biopharmaceuticals. Mobilograms (DT vs *m/z* diagrams) were then calculated for each run and compared for the two experimental conditions. Several differently regulated signals, whose nature is still under investigation, were detected. Among them, a sharp and well-defined group of signals of large molecular weight (*m/z* = 1000–1100) showing a marked upregulation in lesioned striatum was selected for further investigation. The results are reported in Fig. 2. Panels A and B show enlarged mobilograms of the lesioned and control tissues, respectively. The mobilograms were combined to generate the Fusion Map reported in Fig. 2 panel C. This panel reports differentially regulated signals using blue to violet colors for the lesioned tissue and yellow to red colors for the control tissue. These signals (circled by the white line in Fig. 2 panel A) were clearly noticeable due to their marked upregulation (deep blue to violet in the Fusion Map). A complete Fusion Map showing the full *m/z* and RT range is reported in Supplementary Fig. 2 where the black square indicates the investigated signals. On the basis of these ion mobility data, we were able to extract from the total ion current 5 peaks (Fig. 2, panel D) and 5 corresponding *m/z* values (Fig. 2, panel E) that were clearly increased in lesioned tissues relative to controls (named 1–5, details reported in Table 1). Notably, as reported in Fig. 3, panel A, these signals needed to be magnified to become visible from the corresponding total ion current in the 50–1200 *m/z* range. As an example, the mass spectrum of peak 5 (*m/z* = 1042.82) is reported in Fig. 3, panel B (10× magnification). The signals were detected as [M+H]^+^ and [M+NH_4_]^+^ adducts in the positive ion mode, or as [M-H]^-^ adducts in the negative ion mode. One of their key features was an ESI^+^ fragment ion with *m/z* = 310.31 (C_20_H_40_NO, 0.6 ppm mass accuracy). The corresponding ion trace (Fig. 3, panel C) when extracted from the high-energy chromatograms acquired in MS^e^ mode from the control (black trace) and lesioned (red trace) hemispheres, provides an estimate of the relative increase experienced by these lipid species following 6-hydroxy-DA injection. Moreover, in order to obtain a rough indication of the relative abundance of these lipids, and taking into consideration all the differences in structure, ionization and fragmentation, we overlapped the ion current of fragment *m/z* = 310.31 to a well-known fragment ion that is diagnostic of ceramides and glycosylceramides (*m/z* 264.26; double water loss on d18:1 sphingoid base). The corresponding plot (Fig. 3, panel D) gives an indication of the relative abundance of these lipids (red trace) relative to the d18:1 sphingolipid content (black trace). For each of these unknown molecules we calculated the best guess for the molecular formula, on the basis of the accurate mass and isotopic profile. All data are reported in Supporting Table 1. The maximum intensities of these signals ranged between 1 and 3 % of the total ion current measured at the same retention times, explaining why they were not detected by standard PCA analysis or visual inspection of the chromatograms. Surprisingly, neither the accurate masses nor the guessed molecular formulas, when searched against the LIPIDMAPS, METLIN and HMDB databases, returned any hit compatible with the observed isotopic profile, the chromatographic retention time or the adduct type that are normally observed in untargeted lipidomics analysis using our LC-MS/MS setup (Cajka and Fiehn 2014). More importantly, none of the proposed hits was consistent with the high-resolution tandem mass spectra of these lipids. A closer analysis of such data allowed us to tentatively identify the molecules as a subfamily of NAPEs (see Fig. 4 and Supporting Table 2) bearing a polyunsaturated chain (either 22:6 or 20:4) at the *sn*-2 position of the glycerol moiety, and a saturated chain (16:0 or 18:0) at the *N*-acyl position. The latter moiety generates upon fragmentation the *m/z* = 310.31 ion mentioned above. As an example, a detailed interpretation of the ESI^+^ and ESI^−^ MS/MS spectra of lipid 3 (NAPE 18:0-22:6-N18:0, 1058.8170 m/z in ESI^+^) is provided in Fig. 5. Close inspection of the data allowed us to discriminate fragment ion *m/z* = 283.26, which originates from the 18:0 fatty acyl chain from the fragment *m/z* = 283.24 generated by CO_2_ loss from 22:6 fatty acyl chain in *sn*-2 (Song et al. 2007). For two of these signals (*m/z* = 1030.70 and 1014.78), MS/MS analysis revealed the presence of two coeluting isomeric lipids, both carrying 22:6 at *sn*-2 and a reciprocally mass-compensating combination of 18:0 or 16:0 at *sn*-1 and *N*-acyl (for *m/z* = 1030.70) or P-16:0/P-18:0 as *sn*-1 reciprocally compensating 18:0 or 16:0 as N-acyl (for *m/z* = 1014.78). Together, the data identify a group of seven lipids generating five signals detected by IMS analysis. To confirm the compound identification, we synthesized two of these molecules (18:0/22:6/N16:0 and 18:0/22:6/N18:0) and subjected them to LC/MS analysis (Guo et al. 2010; Astarita et al. 2008). The standards showed identical chromatographic and MS/MS behavior as tissue-derived analytes (Supporting Information 1). We also tested the possibility to discriminate the two *sn*-2 fatty acyl chains (20:4 and 22:6) by using IMS. This was done by performing the MS/MS in the trap region and then allowing the fragments to drift in the mobility section of the instrument. The results are reported in the Supplementary Figure 3. The glycerol fragments deriving from the loss of the phosphate group, thus still bearing the *sn*-1 and *sn*-2 acyl chains, were subjected to gas phase drift in the IMS region. Fragments bearing 22:6 as *sn*-2 (from (18:0/22:6/N18:0)) showed a DT value exactly overlapping that of the authentic synthetic standard (Panels A and B) and were clearly distinguishable from the fragment bearing 20:4 (from (18:0/20:4/N18:0), Panel C). According to these data, chain 20:4 is less “bulky” than 22:6 in the gas phase and thus the corresponding fragment ion needs roughly 0.4 milliseconds less to exit the drift region with this setup. We then further explored the overall potential of our IMS protocol by testing it for absolute sensitivity, linearity and selectivity toward NAPEs. The results are reported in the Supporting Data document available for download. Using the synthetic NAPEs as benchmark and the chromatographic setup used for targeted analysis (see the Method section), we compared the absolute sensitivity in MS, IMS and MRM (Multiple Reaction Monitoring) acquisition modes. Although the triple-quad MRM sensitivity exceeded both scan acquisition modes, as expected, IMS enabled a significantly higher sensitivity than MS, with a much higher S/N ratio for a 5 nM calibrator of both synthetic standards (Supplementary Data, Figures 1 and 2). We then compared the instrumental response linearity over more than 3 concentration logs (1–5000 nM): IMS performed significantly better than did MS (Supplementary Data, Figure 3). Finally, we mimicked an untargeted metabolomic experiment by spiking exogenous 18:0/22:6/N17:0 NAPE in brain homogenate at concentrations ranging from 10 ppb to 10 ppm and we acquired the samples in IMS mode. The CMA allowed us to detect the exogenous lipid down to a 1 ppm level (Supplementary Data, Figure 4). At this concentration, PCA analysis and chromatogram overlapping failed to detect the presence of the exogenous lipid in the matrix (Supplementary Data, Figures 5 and 6). Finally, in order to obtain a reliable quantification of these lipids in the brain, we developed a dedicated targeted method using the triple-quadrupole instrument. Lipids were quantified using a calibration curve prepared with synthetic standards, and authentic odd-chain NAPE (18:0-22:6-N17:0) as internal standard. A full validation of this MRM method was also performed, by testing it for linearity, limit of quantification, recovery, matrix effect and extraction efficiency from brain as previously described (Basit et al. 2015). The corresponding results are reported in the Supporting Table 4. After a new tissue extraction, the levels of the seven NAPE species observed were then quantified. The results, shown in Fig. 6, are in good agreement with those previously reported in the literature (Astarita et al. 2008). We conclude that 6-hydroxy-DA lesions elevated NAPE levels in striatal tissue from approximately 10–250 pmol/g to 200–1000 pmol/g. No such change was observed in the SN of the same hemisphere, confirming the highly localized nature of the response.

## Conclusions

The present study shows that signals representing only a small fraction (1–2 %) of the total ion current measured at a given retention time, but that are markedly different between two experimental conditions, can be readily detected by mobilogram comparison even though they are likely to go unnoticed in standard analyses. The reason for this enhanced selectivity lies in the added gas-phase separation that allows for a significantly higher S/N ratio than standard MS. This, together with the highly reproducible LC retention time ensured by UPLC, greatly increases the efficiency in differences detection in metabolomics comparisons. We confirmed the usefulness of this approach by detecting changes in the levels of a scarce class of phospholipids, the NAPEs, in the highly complex lipidome of the brain, where PCA had failed. Although the biological functions of the NAPEs are still poorly understood (Wellner et al. 2013), their biosynthesis is known to be stimulated by tissue damage (Janfelt et al. 2012; Hansen et al. 2000). Thus, when challenged with a complex biological sample, the new method was able successfully to detect elevations in individual lipid species naturally present in the brain at low ppm to ppb levels. This higher level of selectivity can be of great value in neural lipidomics, given the 4–5 log concentration span of the lipid species normally present in brain tissue.

## Electronic supplementary material

Supplementary Data 1 (DOCX 1075 kb)

Supplementary Data 2 (PDF 201 kb)

Supplementary Data 3 (DOCX 470 kb)
