# Supplementary material for: Ion mobility mass spectrometry enhances low-abundance species detection in untargeted lipidomics
Source: Metabolomics. 2016 Feb 8;12:50. doi: 10.1007/s11306-016-0971-3 (PMC4744830; doi:10.1007/s11306-016-0971-3)
Supplement: Supplementary file 1 — Supplementary Data 1 (DOCX 1075 kb) [file 11306_2016_971_MOESM1_ESM.docx]

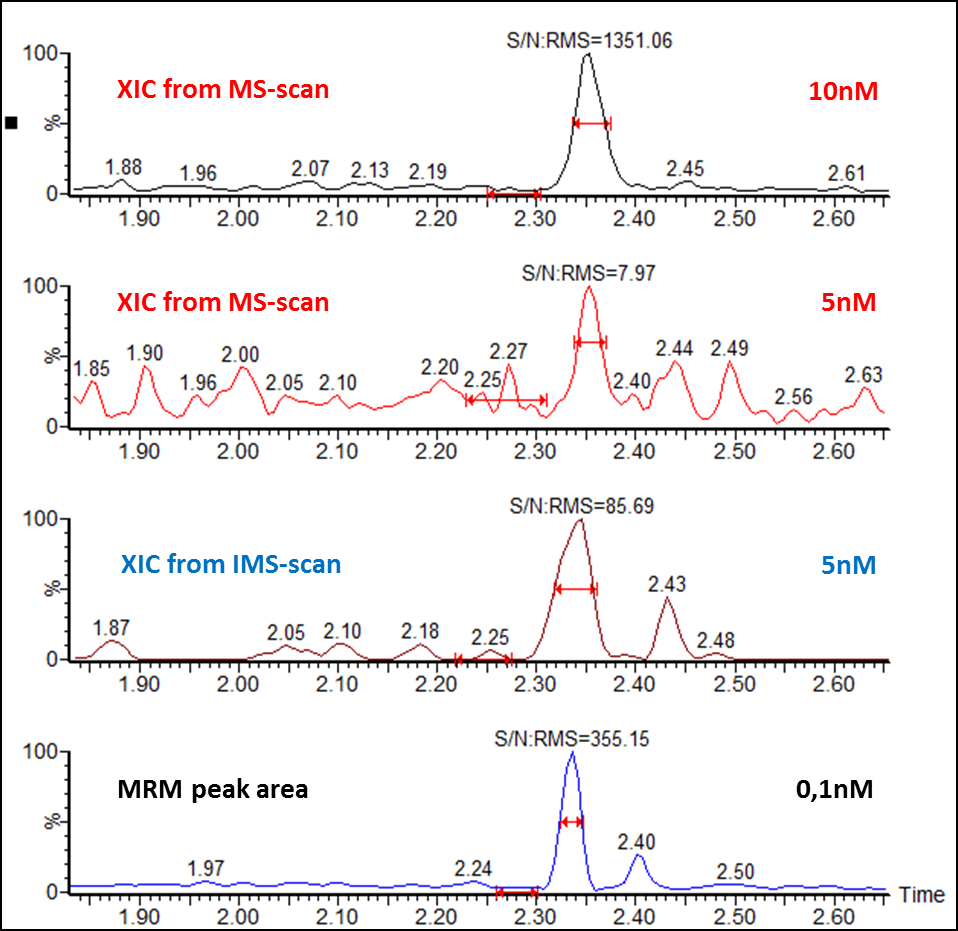


**Figure 1**

Absolute sensitivity comparison for NAPE 16:0/22:6N18:0, in MS scan, IMS scan and targeted MRM modes. IMS shows a significantly higher S/N ratio than MS for the 5nM calibrator (LOQ for IMS scan)


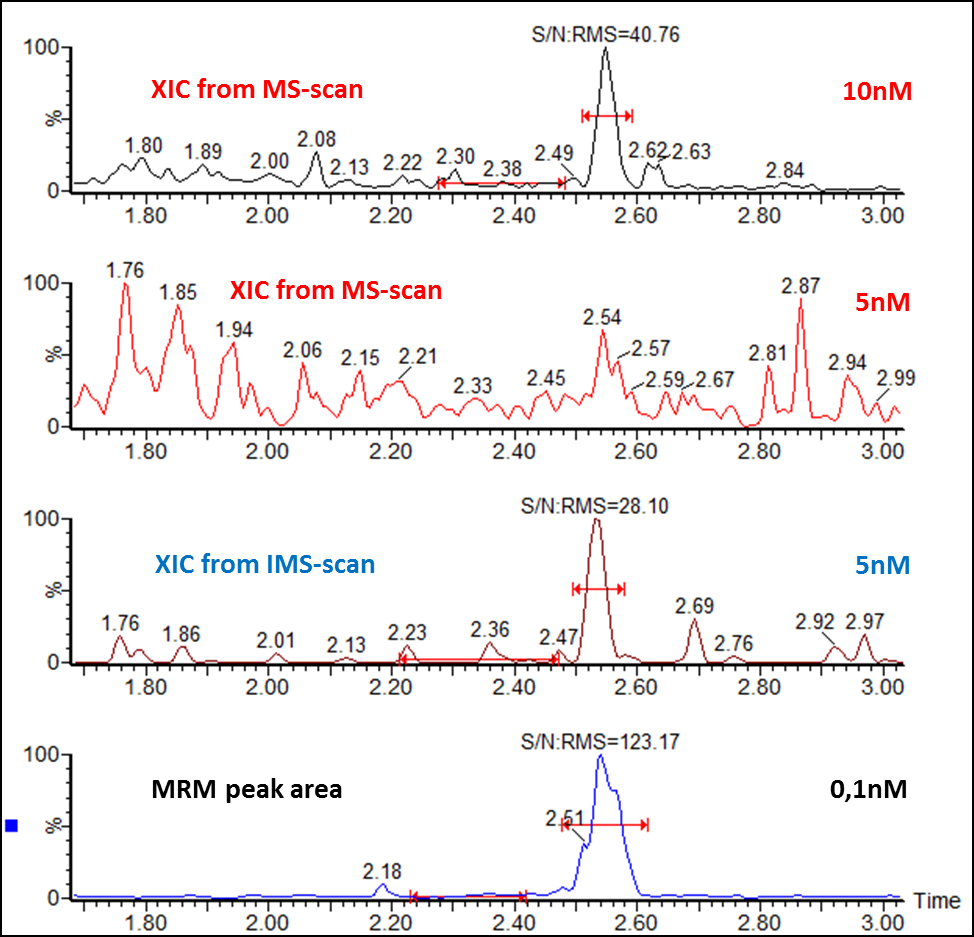


**Figure 2**

Absolute sensitivity comparison for NAPE 18:0/22:6N18:0, in MS scan, IMS scan and targeted MRM modes. IMS shows a significantly higher S/N ratio than MS for the 5nM calibrator (LOQ for IMS scan)

**Figure 3**

Calibration curves of the two synthetic standards prepared over the 1-5000 nM range and analyzed in MS scan and IMS scan acquisition modes. Odd-chain 18:0/22:6/N17:0 NAPE was spiked in the extraction solution to a final 500nM concentration and used for peak area normalization.


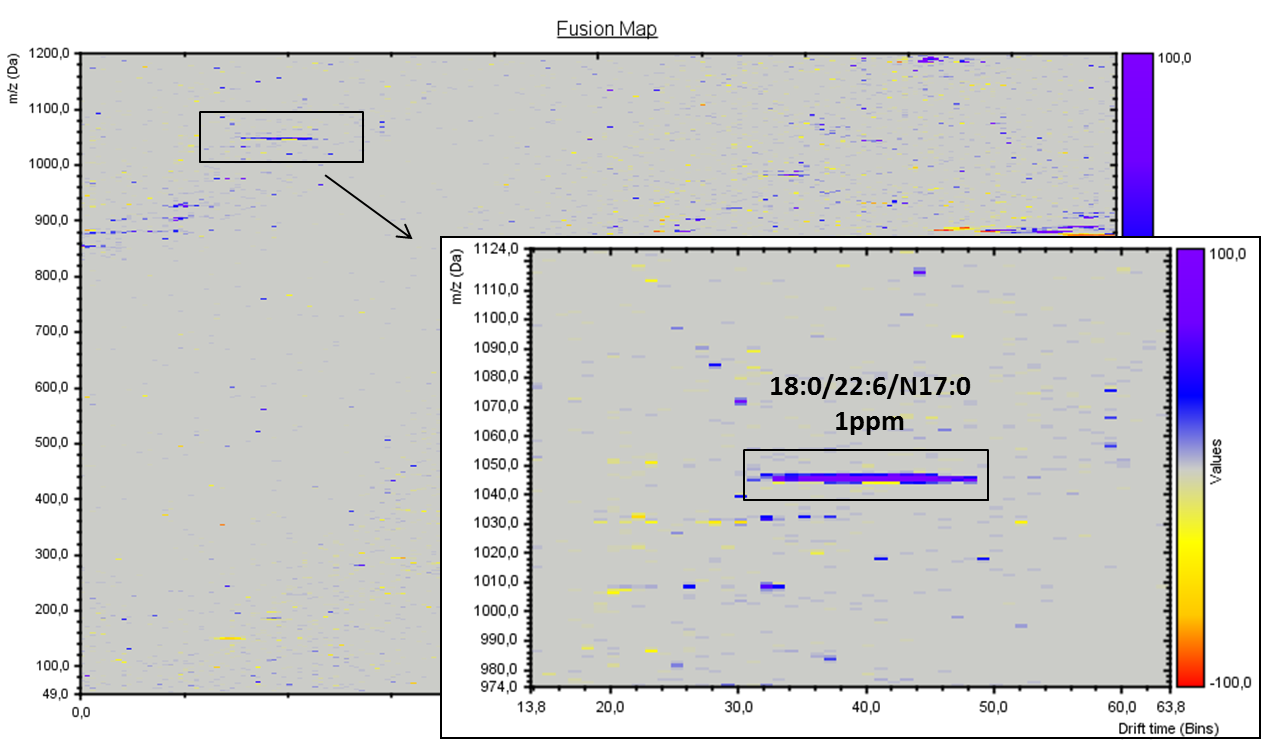


**Figure 4**

Comparative mobilogram analysis of IMS scan acquisitions of naïve brain Vs 1ppm spiking of NAPE 18:0/22:6/N17:0 in naïve brain homogenates. The presence of the exogenous lipid is clearly detectable (blue dots in the black rectangle)

**Figure 5**

Scores Plot from Principal Component Analysis of Naïve brain (black dots) versus a 1ppm spiking of NAPE 18:0/22:6/N17:0 in naïve brain homogenate (red dots). No group separation is observed.


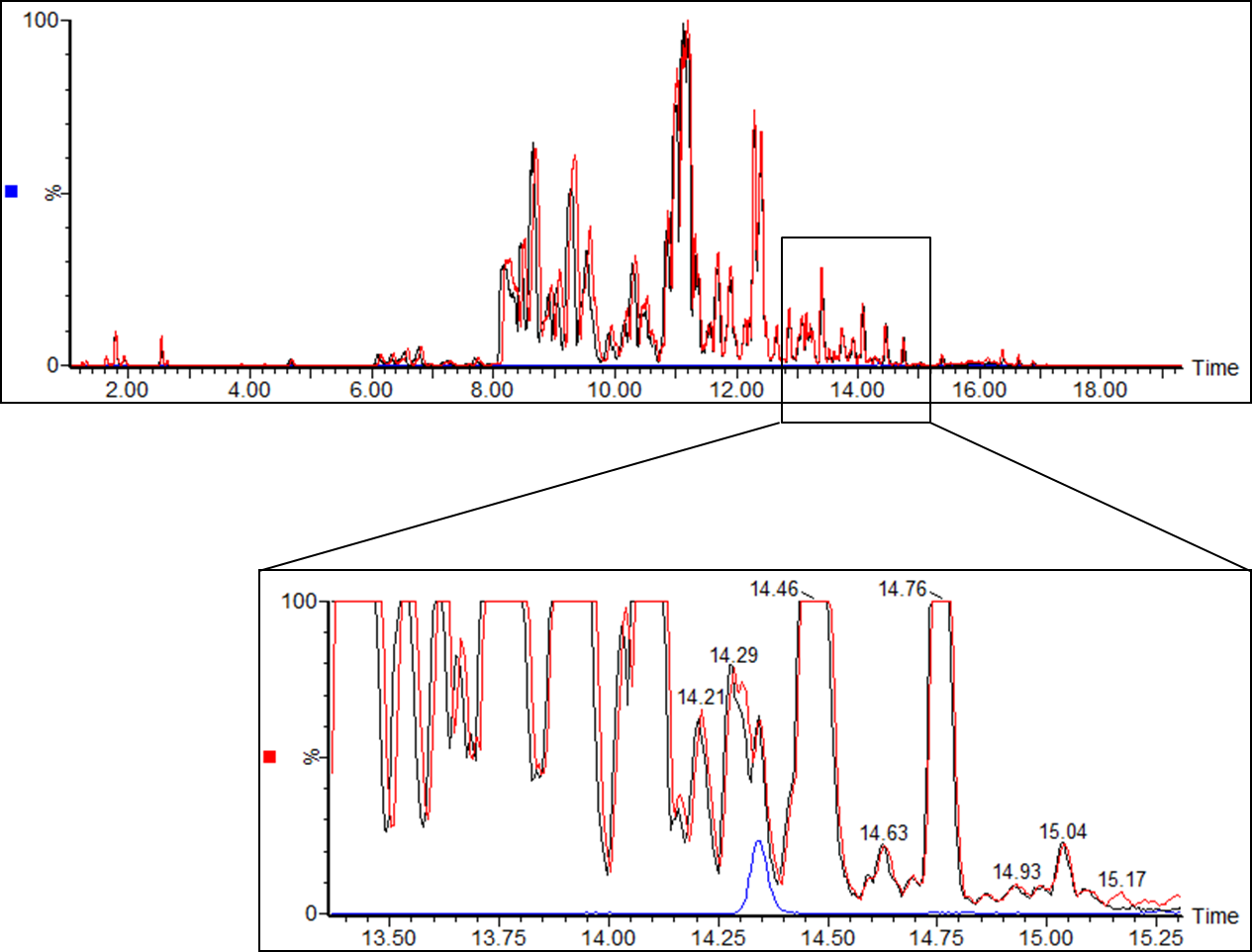


**Figure 6**

Overlapped total ion chromatograms of Naïve brain (black trace) versus a 1ppm spiking of NAPE 18:0/22:6/N17:0 in naïve brain homogenate (red trace). The extracted ion current of the exogenous lipid is reported in blue. At that retention time the two total ion chromatograms are perfectly overlapping.
