# Supplementary material for: Ion mobility mass spectrometry enhances low-abundance species detection in untargeted lipidomics
Source: Metabolomics. 2016 Feb 8;12:50. doi: 10.1007/s11306-016-0971-3 (PMC4744830; doi:10.1007/s11306-016-0971-3)
Supplement: Supplementary file 2 — Supplementary Data 2 (PDF 201 kb) [file 11306_2016_971_MOESM2_ESM.pdf]

Overlapped extracted ion currents

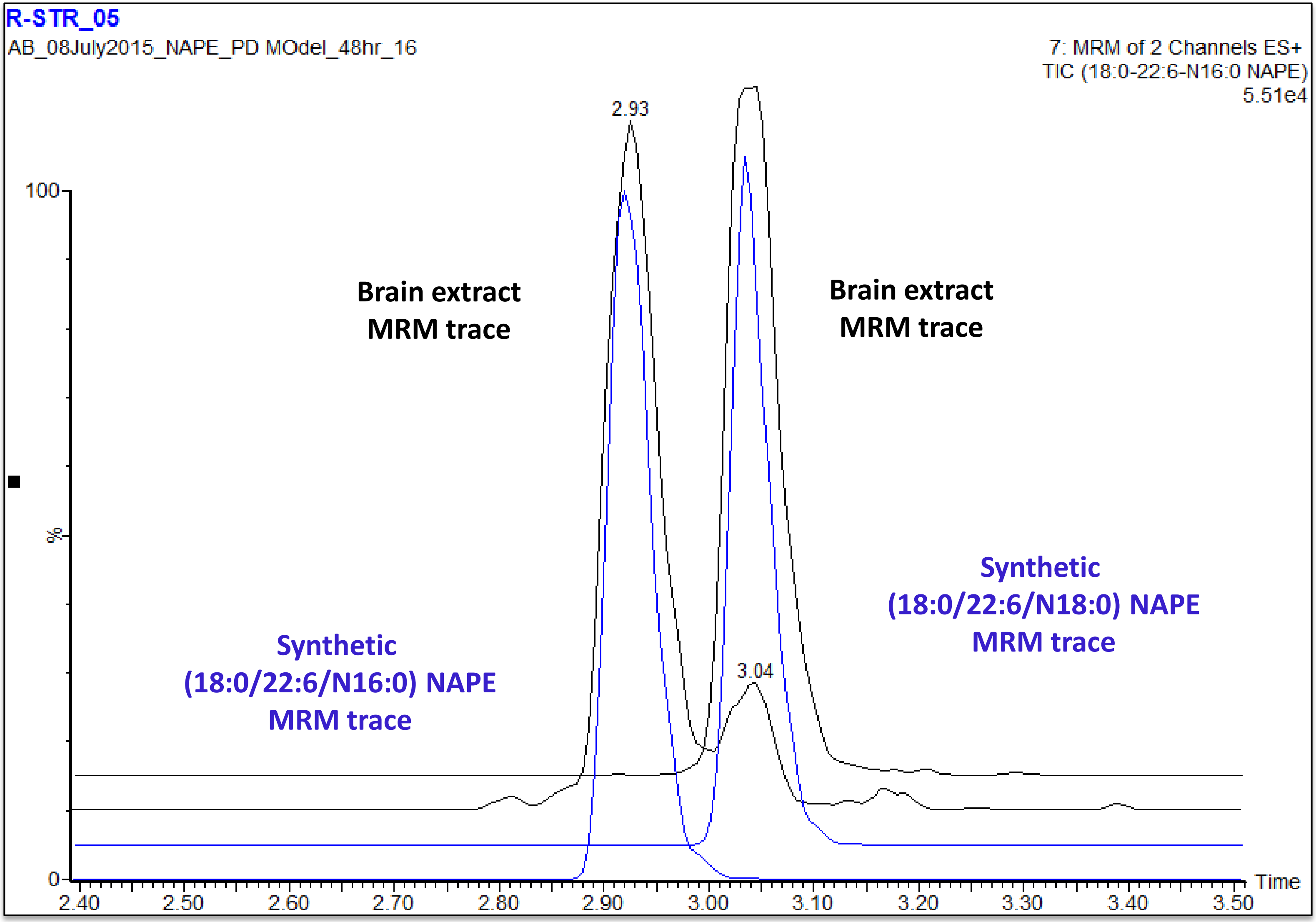

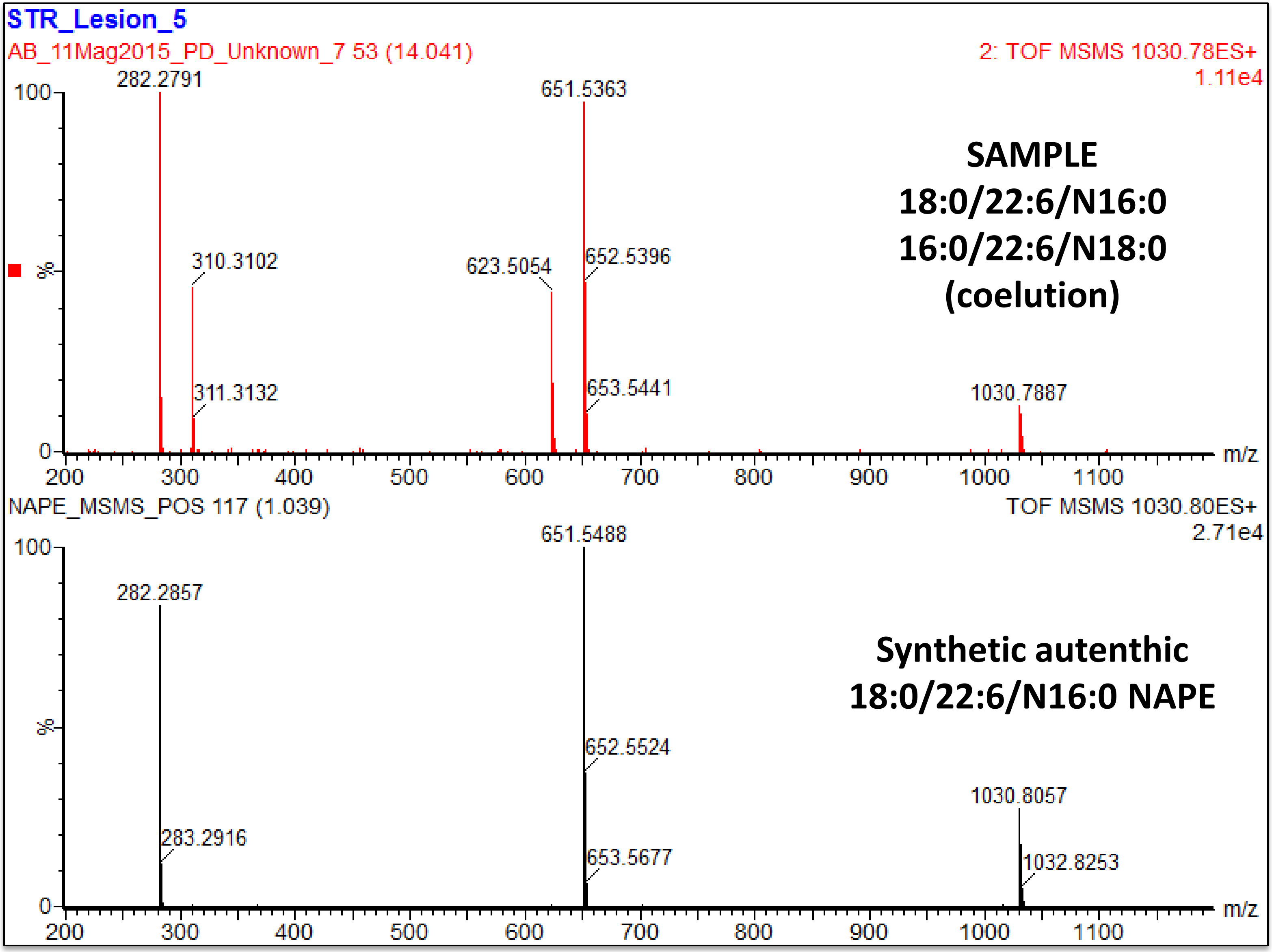
STR_Lesion_5
AB_11Mag2015_PD_Unknown_7 53 (14.041)
2: TOF MSMS 1030.78ES+
1.11e4
282.2791
651.5363
310.3102
623.5054
652.5396
311.3132
653.5441
1030.7887
SAMPLE
18:0/22:6/N16:0
16:0/22:6/N18:0
(coelution)
%
m/z
NAPE_MSMS_POS 117 (1.039)
TOF MSMS 1030.80ES+
2.71e4
651.5488
282.2857
283.2916
652.5524
653.5677
1030.8057
1032.8253
Synthetic autenthic
18:0/22:6/N16:0 NAPE
%
m/z

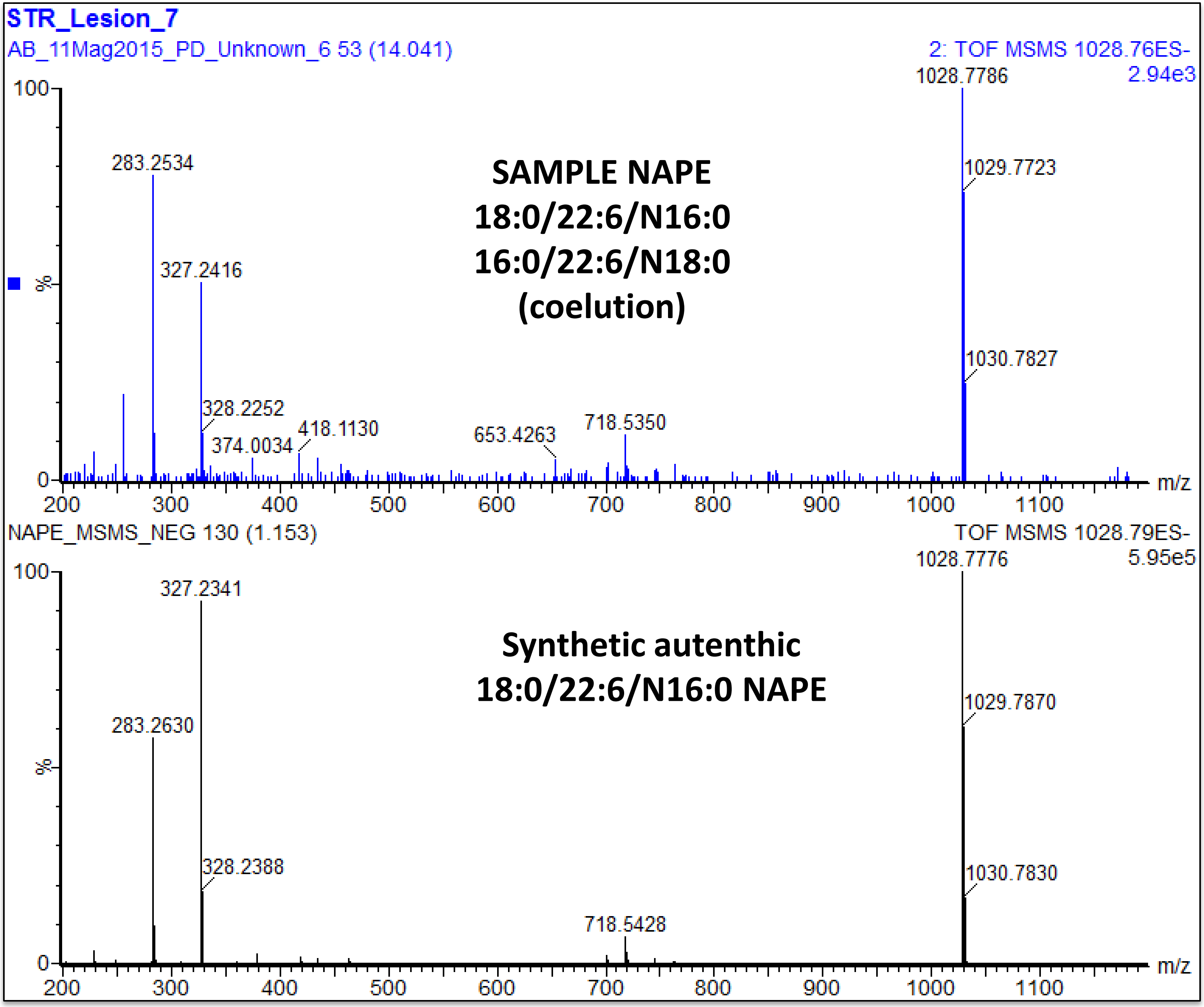

STR_Lesion_7
AB_11Mag2015_PD_Unknown_6 53 (14.041)
2: TOF MSMS 1028.76ES-
2.94e3
1028.7786
283.2534
327.2416
1029.7723
1030.7827
328.2252
418.1130
374.0034
653.4263
718.5350
SAMPLE NAPE
18:0/22:6/N16:0
16:0/22:6/N18:0
(coelution)
%
m/z
NAPE_MSMS_NEG 130 (1.153)
TOF MSMS 1028.79ES-
5.95e5
1028.7776
327.2341
283.2630
328.2388
718.5428
1029.7870
1030.7830
Synthetic autenthic
18:0/22:6/N16:0 NAPE
%
m/z

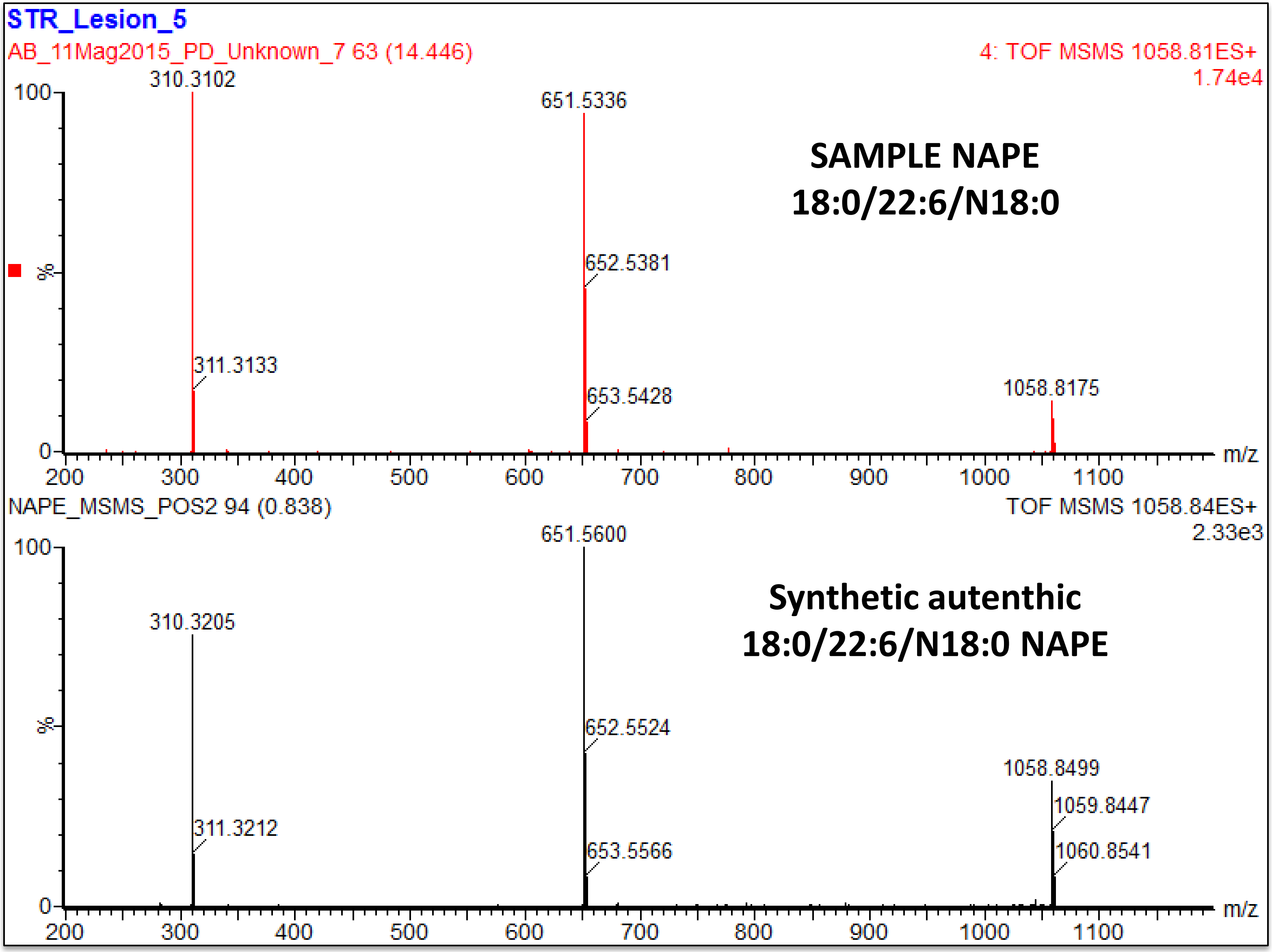
STR_Lesion_5
AB_11Mag2015_PD_Unknown_7 63 (14.446)
4: TOF MSMS 1058.81ES+
1.74e4
310.3102
651.5336
SAMPLE NAPE
18:0/22:6/N18:0
652.5381
311.3133
653.5428
1058.8175
100
%
0
200
300
400
500
600
700
800
900
1000
1100
m/z
NAPE_MSMS_POS2 94 (0.838)
TOF MSMS 1058.84ES+
2.33e3
651.5600
Synthetic autenthic
18:0/22:6/N18:0 NAPE
310.3205
652.5524
1058.8499
1059.8447
311.3212
653.5566
1060.8541
100
%
0
200
300
400
500
600
700
800
900
1000
1100
m/z

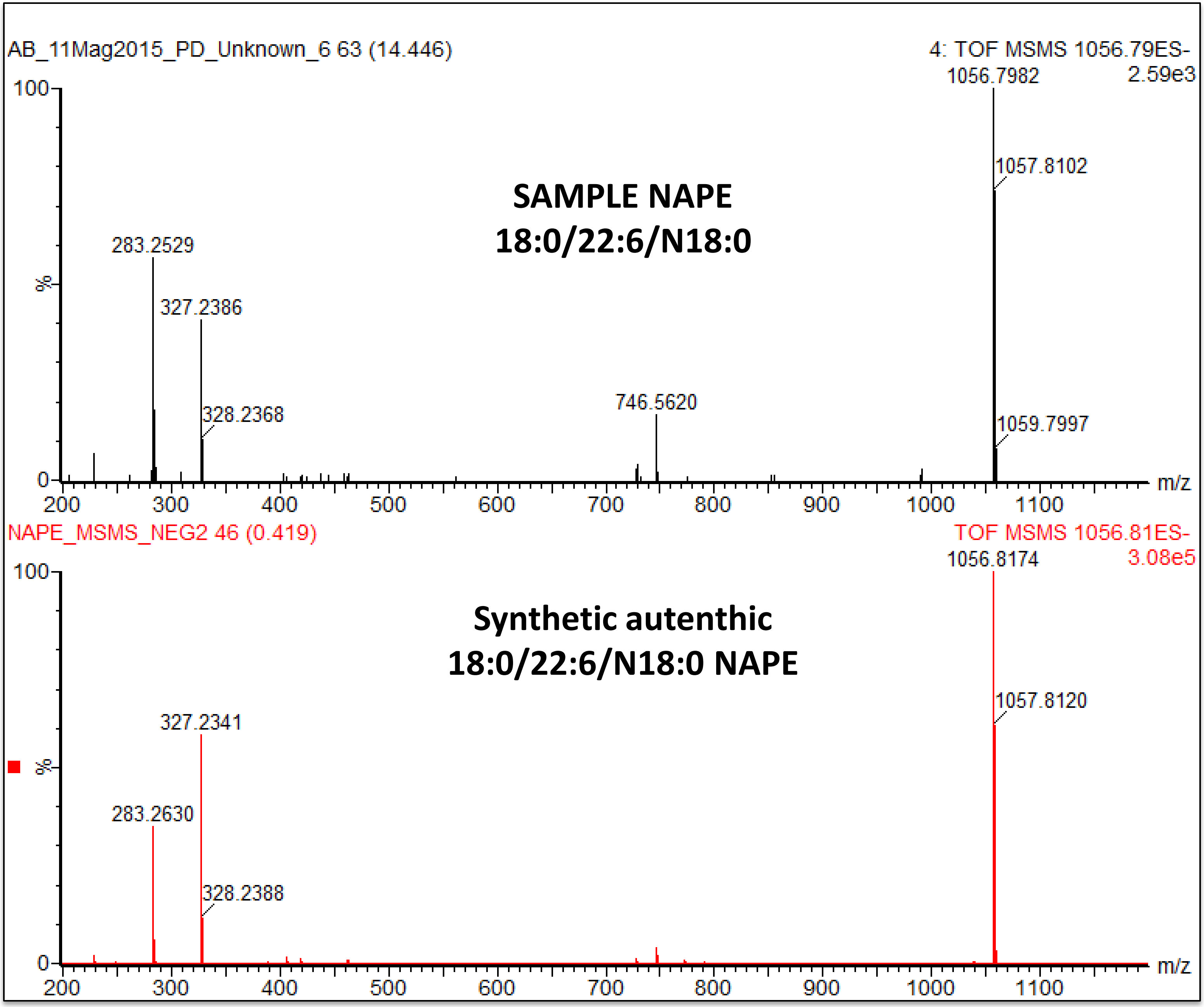
AB_11Mag2015_PD_Unknown_6 63 (14.446)
4: TOF MSMS 1056.79ES-
2.59e3
SAMPLE NAPE
18:0/22:6/N18:0
1056.7982
1057.8102
283.2529
327.2386
328.2368
746.5620
1059.7997
100
%
0
200
300
400
500
600
700
800
900
1000
1100
m/z
NAPE_MSMS_NEG2 46 (0.419)
TOF MSMS 1056.81ES-
3.08e5
Synthetic autenthic
18:0/22:6/N18:0 NAPE
1056.8174
1057.8120
327.2341
283.2630
328.2388
100
%
0
200
300
400
500
600
700
800
900
1000
1100
m/z
