# Supplementary material for: Ion mobility mass spectrometry enhances low-abundance species detection in untargeted lipidomics
Source: Metabolomics. 2016 Feb 8;12:50. doi: 10.1007/s11306-016-0971-3 (PMC4744830; doi:10.1007/s11306-016-0971-3)
Supplement: Supplementary file 3 — Supplementary Data 3 (DOCX 470 kb) [file 11306_2016_971_MOESM3_ESM.docx]

**Supplementary Figure 1**

Intrastriatal injection of 6-hydroxy-DOPA induces microglia activation. Immunofluorescence of Iba1 (green) revealed resting highly ramified microglia within the dorsal striatum of control animals (A) In contrast, animals injected with 6-hydroxy-DOPA 48 hours after the injection showed activated microglia with shorter and thicker processes and larger cell bodies (B). Calibration bar=30 microns.


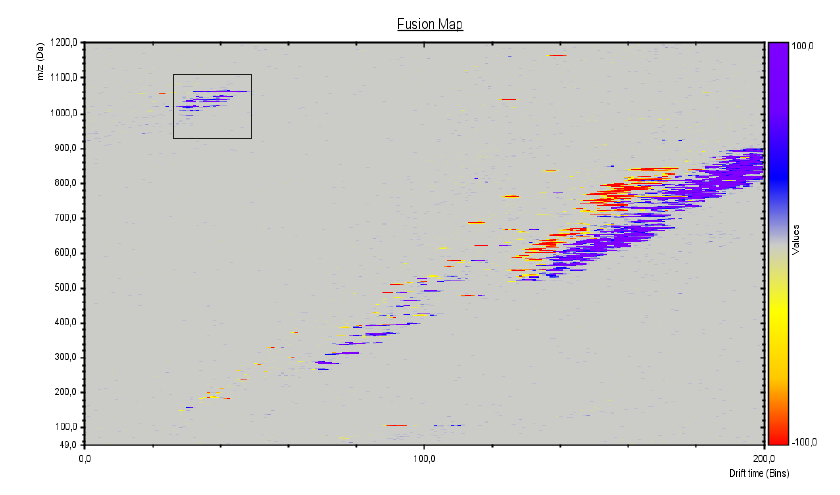


**Supplementary Figure 2**

Full m/z, RT range Fusion map obtained by overlapping control (yellow to red) and lesion (blue to violet) tissue mobilograms. The well-defined family of signals (1000-1100 m/z) investigated in this study is highlighted with the black square and it is enlarged in Figure 2, Panel C.


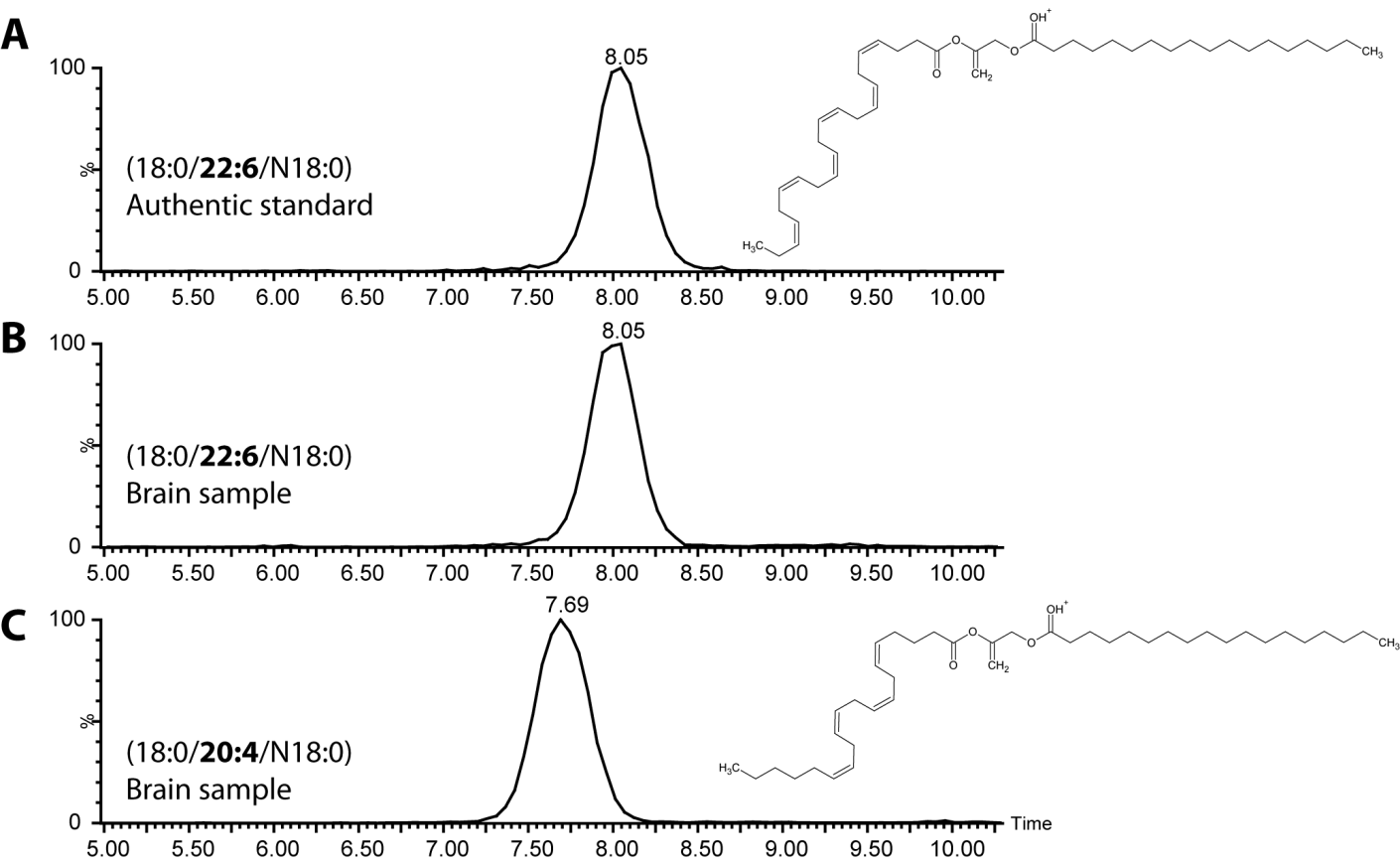


**Supplementary Figure 3**

Drift time profiles for (18:0/**22:6**/N18:0) from synthetic standard (Panel A) and brain sample (Panel B) (sn-1/sn-2 fragment ion at 651.53 m/z) and for (18:0/**20:4**/N18:0) from brain sample (Panel C) (sn-1/sn-2 fragment ion at 627.53 m/z). The impact of 22:6 or 20:4 acyl chain at sn-2 position on gas phase ion mobility can be clearly detected. IMS arrival times are indicated on the top of the peak. Timescale is in milliseconds.

|  |  | **Observed Adducts** | |  | **ESI+** | |  |  |  |
| --- | --- | --- | --- | --- | --- | --- | --- | --- | --- |
| **Unknown** | **Retention Time (min)** | **[M+H]^+^** | **[M-H]^-^** | **Tentative**  **Molecular Formula** | **Theoretical** | **ppm** | **Max XIC** | **TIC @ RT** | **% of TIC** |
| **1** | 14,07 | 1030,7852 | 1028,7653 | C61H108NO9P | 1030,7839 | 1,3 | 18894 | 7,92E+05 | 2% |
| **2** | 14,30 | 1014,7893 | 1012,7703 | C61H108NO8P | 1014,7891 | 0,2 | 15211 | 6,69E+05 | 2% |
| **3** | 14,49 | 1058,817 | 1056,7948 | C63H112NO9P | 1058,8153 | 1,6 | 17818 | 5,43E+05 | 3% |
| **4** | 14,63 | 1034,8204 | 1032,8196 | C61H112NO9P | 1034,8153 | 4,9 | 11755 | 5,38E+05 | 2% |
| **5** | 14,70 | 1042,8252 | 1040,8011 | C63H112NO8P | 1042,8204 | 4,6 | 15057 | 1,29E+06 | 1% |

**Supporting Table 1: Upregulated signals detected by IMS data comparison.**

| **Analyte** | **Molecular Formula** | **[M+H]^+^** | ***Sn*-1** | ***Sn*-2** | **N-Acyl** |
| --- | --- | --- | --- | --- | --- |
| **1** | C61H108NO9P | 1030,7839 | 16:0 or 18:0 | 22:6 | 16:0 or 18:0 |
| **2** | C61H108NO8P | 1014,7891 | P-18:0 or P-16:0 | 22:6 | 16:0 or 18:0 |
| **3** | C63H112NO9P | 1058,8153 | 18:0 | 22:6 | 18:0 |
| **4** | C61H112NO9P | 1034,8153 | 18:0 | 20:4 | 18:0 |
| **5** | C63H112NO8P | 1042,8204 | P-18:0 | 22:6 | 18:0 |

**Supporting Table 2: Fatty acyl chain composition of NAPEs derived from mouse striatum.**

| **Sr. No.** | **sn-1, sn-2 NAPEs** | **MRM transitions** |
| --- | --- | --- |
| **1** | P18:0-22:6-N16:0 | 1014.8🡪282.3/635.5 |
| **2** | P16:0-22:6-N18:0 | 1014.8🡪310.3/607.5 |
| **3** | 18:0-22:6-N16:0 | 1030.8🡪282.3/651.5 |
| **4** | 16:0-22:6-N18:0 | 1030.88🡪310.3/623.5 |
| **5** | 18:0-20:4-N18:0 | 1034.8🡪310.3/627.5 |
| **6** | P18:0-22:6-N18:0 | 1042.8🡪310.3/635.5 |
| **7** | 18:0-22:6-N18:0 | 1058.8🡪310.3/651.5 |
| **IS** | 18:0-22:6-N17:0 | 1044.6🡪296.4/651.5 |

**Supporting Table 3:** MRM transitions used for the targeted quantification of NAPEs

|  | **Average (N=3)** | **CV% (N=3)** |
| --- | --- | --- |
| **LOQ** | 0.05 nM | 3 |
| **Recovery** | 107 | 9 |
| **Matrix Effect** | 27% (Suppression) | 6 |
| **Extraction Efficiency** | 75 | 13 |
| **Linearity (R2)** | 0.9969 | 0.3 |
|  | | |

**Supporting Table 4:** Validation of the targeted MRM method. Exogenous 18:0/22:6/N17:0 NAPE was spiked in naive brain homogenate and extracted (N=3). Limit of quantification, recovery, matrix effect, extraction efficiency were evaluated using the calibration curve showed in the inset (0.05 to 50nM, R^2^=0.9969).
